# Supplementary material for: No Evidence Known Viruses Play a Role in the Pathogenesis of Onchocerciasis-Associated Epilepsy. An Explorative Metagenomic Case-Control Study
Source: Pathogens. 2021 Jun 22;10(7):787. doi: 10.3390/pathogens10070787 (PMC8308762; doi:10.3390/pathogens10070787)
Supplement: Supplementary file 1 [file pathogens-10-00787-s001.zip › pathogens-1235243-supplementary.pdf]

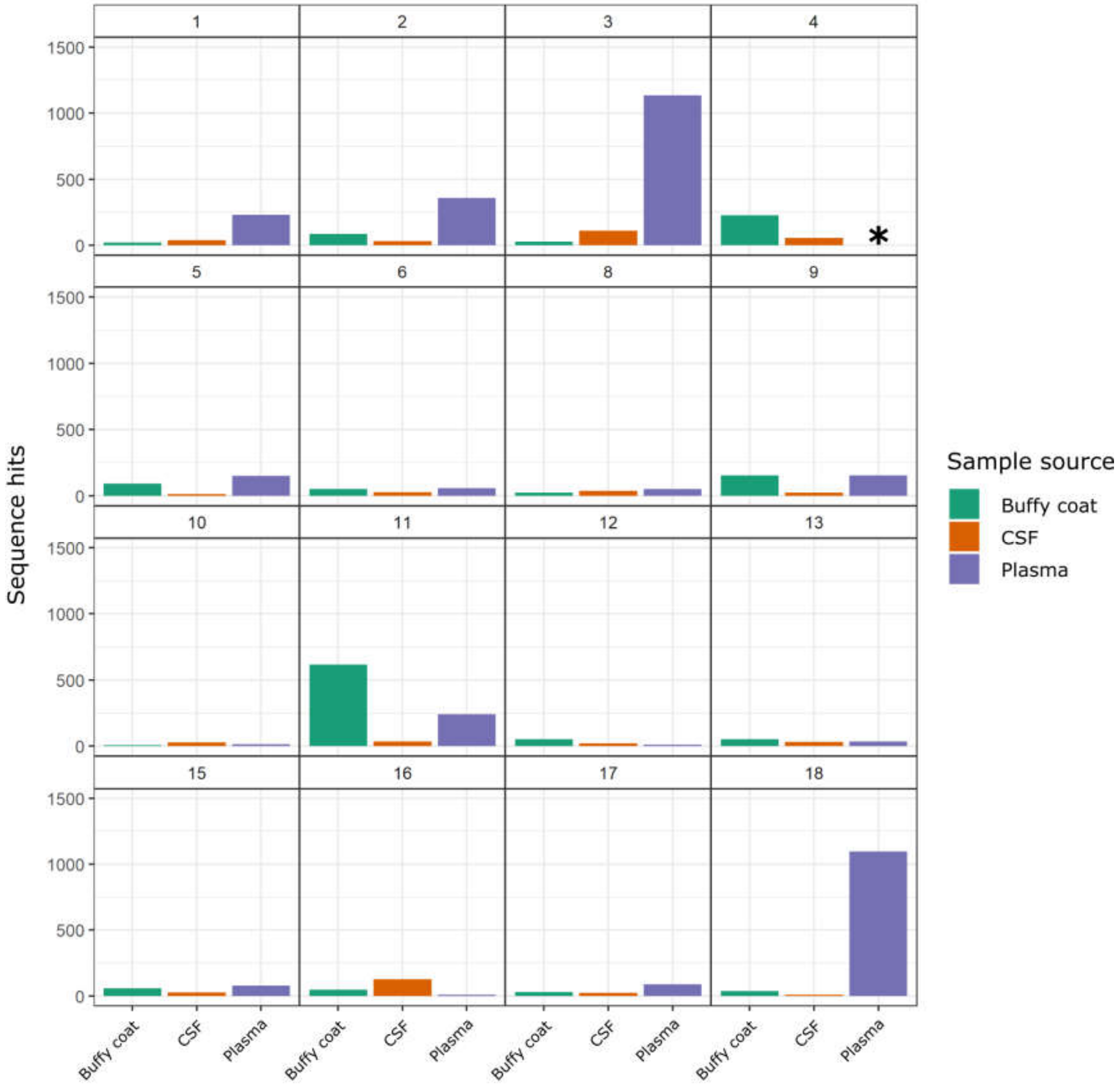

Supplementary Figure S1: Virus sequence hit counts are shown for each case individual, separated and colored by sample source. *Plasma hits for patient #4 are not shown as they exceeds the scale for this graph.

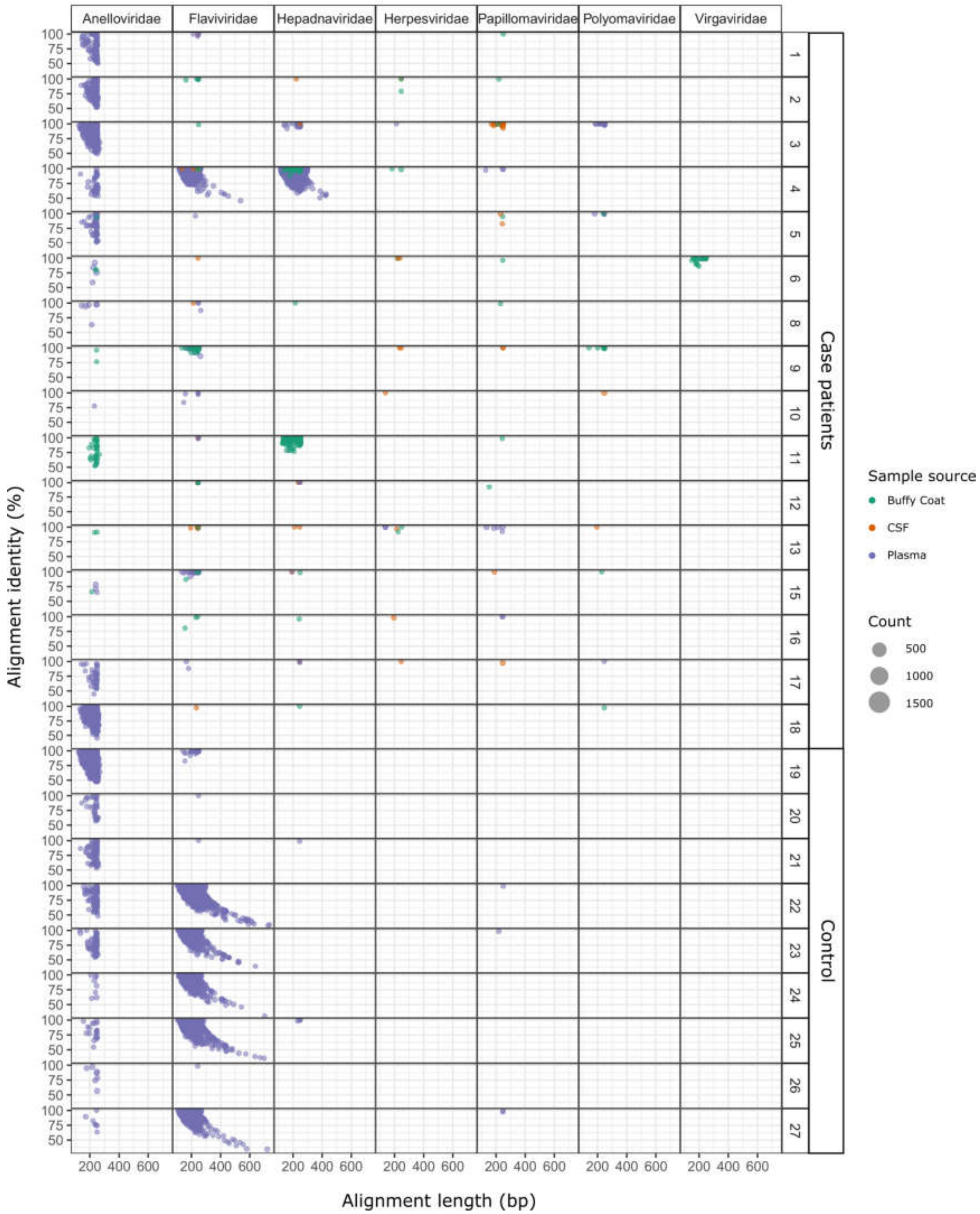

Supplementary Figure S2: Per-patient hit sequences for seven viral families. Hit sequences are separated by individual, colored by sample source, and scaled according to count. Case and control groups are indicated.
